# Total carbon accumulation in a tropical forest landscape

**DOI:** 10.1186/1750-0680-7-12

**Published:** 2012-12-19

**Authors:** Carlos A Sierra, Jorge I del Valle, Hector I Restrepo

**Affiliations:** 1 Max Planck Institute for Biogeochemistry, Hans-Knöll-Str. 10, Jena, 07745, Germany; 2 Research Center on Ecosystem and Global Change Carbono & Bosques, Medellín, Colombia; 3 Universidad Nacional de Colombia Sede Medellín, Medellín, Colombia

## Abstract

**Background:**

Regrowing tropical forests worldwide sequester important amounts of carbon and restore part of the C emissions emitted by deforestation. However, there are large uncertainties concerning the rates of carbon accumulation after the abandonment of agricultural and pasture land. We report here accumulation of total carbon stocks (TCS) in a chronosequence of secondary forests at a mid-elevation landscape (900-1200 m asl) in the Andean mountains of Colombia.

**Results:**

We found positive accumulation rates for all ecosystem pools except soil carbon, which showed no significant trend of recovery after 36 years of secondary succession. We used these data to develop a simple model to predict accumulation of TCS over time. This model performed remarkably well predicting TCS at other chronosequences in the Americas (Root Mean Square Error < 40 Mg C ha^-1^), which provided an opportunity to explore different assumptions in the calculation of large-scale carbon budgets. Simulations of TCS with our empirical model were used to test three assumptions often made in carbon budgets: 1) the use of carbon accumulation in tree aboveground biomass as a surrogate for accumulation of TCS, 2) the implicit consideration of carbon legacies from previous land-use, and 3) the omission of landscape age in calculating accumulation rates of TCS.

**Conclusions:**

Our simulations showed that in many situations carbon can be released from regrowing secondary forests depending on the amount of carbon legacies and the average age of the landscape. In most cases, the rates used to predict carbon accumulation in the Americas were above the rates predicted in our simulations. These biome level rates seemed to be realistic only in landscapes not affected by carbon legacies from previous land-use and mean ages of around 10 years.

## Background

Land-use change is a complex phenomenon within the tropical biome [1-3]. Deforestation, one component of land-use change, has received a considerable amount of attention due to important consequences on biological diversity, carbon, and nutrient cycling. Less studied however, is the process of forest regrowth, which occurs when land under a given anthropogenic use is abandoned and a new forest establishes. In some cases the new forest recovers important ecological attributes of the original one such as structure, function, and composition [4,5].

At the continental level, regrowing tropical forests compensate part of the carbon emissions from tropical deforestation [6]. After agricultural and pasture land is abandoned, new vegetation quickly establishes, with the highest rates of aboveground biomass accumulation within the first 15 years [4], and recovering to levels similar to old-growth forests in a time frame between 80 to 100 years [5,7].

Pan et al. [6] recently estimated a global carbon sink from re-growing tropical forests, on average, on the order of 1.65 ± 0.71 Pg C yr^-1^. These authors however, report that their estimate is subject to large uncertainties. Because it is based on published data on aboveground biomass accumulation, one important source of uncertainty is the unknown rates of accumulation in other ecosystem carbon pools.

Estimates of forest carbon accumulation rates seldom include pools other than aboveground biomass due to the difficulties in sampling components such as coarse and fine roots, fine litter and coarse woody debris, and soil carbon. Measurement of carbon accumulation in these different pools would help to reduce bias significantly.

In addition to uncertainties and bias related to the pools included in estimating tropical forest regrowth, there is uncertainty in the assumptions of landscape configuration and legacies from previous land-use [8]. Forest regrowth varies considerably over forest succession, with high accumulation rates in the first stages and declining over time [4,9]. Secondary forest landscapes in the tropics are usually heterogeneous [10,11], so it is therefore uncertain whether one single average accumulation rate can characterize well this heterogeneity in landscape ages.

An additional source of uncertainty are carbon legacies from previous land use [8]. Depending on the type and the time since the previous land use, there could be important effects of carbon legacies as well. A carbon legacy is a given amount of dead carbon remaining from the previous land use that is subject to decomposition [9,12,13]. Examples of carbon legacies include: dead organic debris after hurricanes and large tropical storms [14-16], coarse woody debris after logging operations [17], or residues left after crop harvesting [18], among others. Carbon legacies can have important effects on ecosystem carbon fluxes, switching its behavior from carbon sinks to sources [13,19].

The main objective of this study was to estimate rates of total carbon accumulation from a chronosequence of regrowing tropical forests in the Porce region of Colombia, derive a predictive empirical model, and compare its predictions with estimations of tropical forest regrowth used for large-scale carbon budgets. In addition, we were interested in exploring different assumptions often made in predicting continental level carbon accumulation. In particular, 1) the use of carbon accumulation in tree aboveground biomass as a surrogate for total carbon accumulation rates, 2) the implicit (versus explicit) representation of carbon legacies, and 3) the omission of landscape age in calculating C accumulation rates.

## Results

### Carbon accumulation in different pools

**Figure 1:**
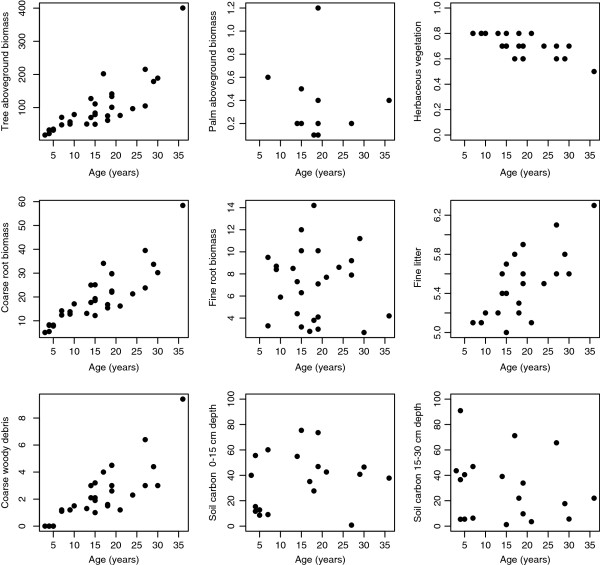
**Carbon accumulation with successional age in total tree aboveground biomass, palms, herbaceous vegetation, coarse roots, fine roots, fine litter, coarse woody debris, and soil carbon between 0-15 and 15-30 cm depth.** All units in Mg DW ha^-1^, except soil carbon in Mg C ha^-1^.

Carbon storage increased along the 36 year chronosequence for most but not all of the measured pools. Tree aboveground biomass, coarse roots, fine litter, and coarse woody debris showed positive trends of accumulation, while palm aboveground biomass, herbaceous vegetation, fine root biomass, and soil carbon showed no trend over time (Figure 1).

The highest amount of carbon was accumulated in tree aboveground biomass, followed by coarse root biomass, and coarse woody debris. Palm aboveground biomass and herbaceous vegetation contributed the smallest amount of carbon (Figure 1).

**Figure 2:**
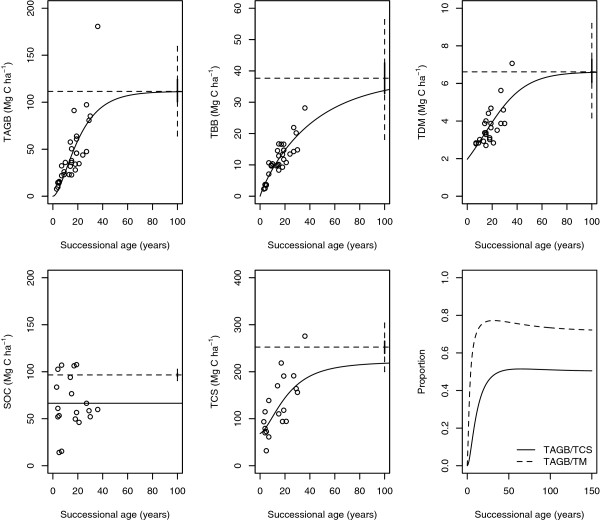
**Best fit models (continuous lines) based on measured (circles) ecosystem carbon pools: total aboveground biomass (*TAGB*), total belowground biomass (*TBB*), total dead mass (*TDM*), soil carbon to 30 cm depth (*SOC*), and total carbon stocks (*TCS*).** Mean and variance of carbon stocks in undisturbed forests of the region are represented with a dotted line and with a boxplot on the right side of each panel.

When aggregated in major ecosystem carbon pools, total aboveground biomass was consistently the highest contribution to total carbon stocks compared to all other pools (∼ 50%), but the relative contribution of aboveground biomass changed significantly during the successional sequence (Figure 2). During the first 5 to 7 years of forest succession, total aboveground biomass contributes less than 20% to TCS, but this contribution changes fast and reaches a maximum at about 40 years.

Soil carbon in the first 30 cm represents the second most important ecosystem carbon pool, with an average of 66.5 ± 28.1 Mg C ha^-1^, but in this landscape it does not present any accumulation trend. In fact, the stocks of carbon in these secondary forest soils are significantly lower than in soils of primary forests and showed no sign of recovery (Figure 2).

### Model fits to aggregated pools

The von Bertalanffy model (equation 4) provided the best statistical fits for total aboveground biomass and total belowground biomass, while the logistic equation (equation 5) provided the best fit to total dead mass (Table 1, Figure 2). However, the addition of the parameter *β*_0_ in the von Bertalanffy model did not improve the statistical fits, and for this reason it was not included in the final models.

**Table 1:** **Best parameter estimates of non-linear regression models applied to total aboveground biomass (*TAGB*), total belowground biomass (*TBB*) and total dead mass (*TDM*)**

**Equation**	**Parameter estimate**	**Standard error**	***p*-value**
$$\begin{array}{c} \text{TAGB}=\\ 111.51\left(1,-,\exp\right)\\ \left(-,\beta_{1},*,t\right){\left(\right)}^{\beta_{2}} \end{array}$$	*β*_1 _= 0*.*064	0.021	0.004
	*β*_2 _= 1*.*964	0.851	0.028
$\begin{array}{c} \text{TBB}=\\ 37.665\left(1,-,\exp\right)\\ \left(-,\beta_{1},*,t\right){\left(\right)}^{\beta_{2}} \end{array}$	*β*_1 _= 0*.*022	0.005	< 0*.*001
	*β*_2 _= 0*.*897	0.149	< 0*.*001
$\begin{array}{c} \text{TDM}=\\ \frac{6.615}{\left(1,+,\beta_{1},\exp,\left(\beta_{2},t\right)\right)} \end{array}$	*β*_1 _= 2*.*363	0.594	< 0*.*001
	*β*_2 _= -0*.*062	0.014	< 0*.*001

*t* represents the forest age since land-use abandonment.

A model for total carbon stocks was then obtained analytically, summing each pool algebraically as in equation (2), which results in

(1) $$\begin{array}{lll} \;\text{TCS}= & 66.452+111.51{\left(1,-,\exp,\left(-,0,.,064,*,t\right)\right)}^{1.964}\; & \;\\ & +37.665{\left(1,-,\exp,\left(-,0,.,022,*,t\right)\right)}^{0.897}\;\\ & +\frac{6.615}{\left(1+2.363\exp(-0.062t)\right)}.\; & \; \end{array}$$

The first term of this equation is the average soil carbon measured in the secondary forests, which in this case, does not accumulate over time.

### Simulations

**Figure 3:**
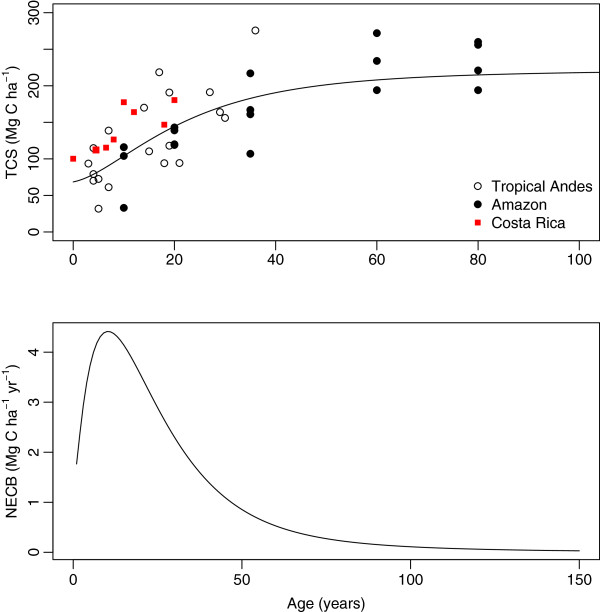
**Total carbon stocks (*TCS*, top) and net ecosystem carbon balance (*NECB*, bottom) with successional age.** Open circles in top panel represent measurements obtained in this study, filled circles represent independent data reported by [21] for an Amazon tropical forest, and squares independent data from a lowland forest in Costa Rica [22]. Lines represent predictions by the fitted model (equation 1).

The empirical model of total carbon accumulation derived for the forests of the Porce region (equation 1), performed surprisingly well predicting carbon accumulation in other chronosequences of total carbon accumulation in the Amazon basin [20,21] and in lowland forest of Costa Rica [22]. The root mean squared error (RMSE) estimated an average deviation between model predictions and observations as 35.1 and 40.5 Mg C ha^-1^ for the Amazon and Costa Rican datasets, respectively. The model was able to predict the trend of rapid carbon accumulation during the initial years of forest succession and the subsequent decline of accumulation rates at later ages that have been observed in other forests [4,9,13,19,23] (Figure 3). The maximum rate of carbon accumulation, 4.4 Mg C ha^-1^yr^-1^, was reached at an average age of 9 years.

#### Effects of carbon legacies

The effects of carbon legacies on TCS were assessed by performing simulations in which different levels of C legacies were left in situ at the beginning of the successional sequence. Carbon legacies decomposed over time following an exponential model using a dataset of decomposition rates for tropical trees [24,25].

**Figure 4:**
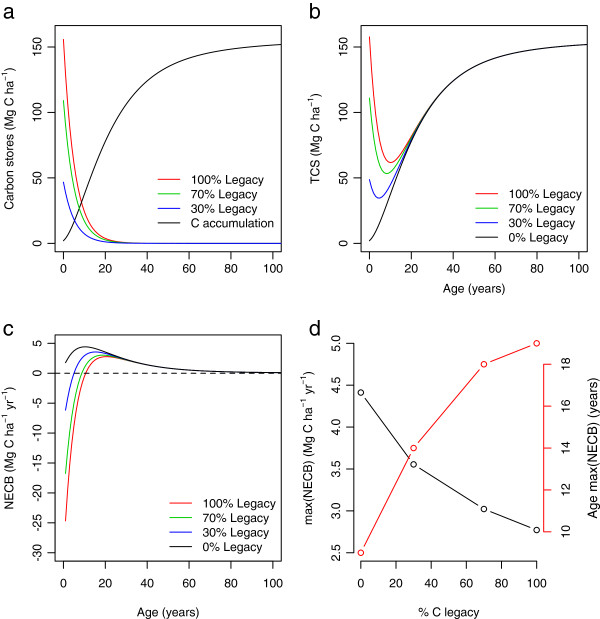
**Effects of carbon legacies on total carbon accumulation and net ecosystem carbon balance (*NECB*). a**) Temporal behavior of legacy carbon (colored lines) and new carbon accumulation (black line). **b**) Total carbon stocks (*TCS*) under different assumptions of carbon legacies. **c**) Net ecosystem carbon balance (*NECB*) under different assumptions of C legacies. **d**) Effects of C legacies on the maximum accumulation rates (max(*NECB*)), and the age at which this rate is reached (Age max(*NECB*)). % C legacy represents the percentage of C left in situ to decompose from the total carbon mass (TM) present in undisturbed forests. Decomposition of C legacies according to decomposition rates reported by [24].

Our simulations showed that carbon legacies from previous land use can persist in regrowing tropical forests, on average, for up to 30 years (Figure 4a). The higher the amount of legacy carbon from the previous land use, the more persistent its effect over time.

In the presence of carbon legacies, the total amount of carbon in an ecosystem declines during the first years of secondary succession. This trend contrasts with ecosystems with no legacies, where carbon always accumulates during the early years (Figure 4b).

In terms of carbon fluxes, net ecosystem carbon balance (*NECB*) is always negative in the early stages of succession when carbon legacies are present, until forest regrowth compensate C release (Figure 4c).

Carbon legacies also introduce time-lags in the age of maximum carbon accumulation rates. Without carbon legacies, the maximum rates occurred at year 9 in our empirical model with no legacies. As carbon legacies increased to 30, 70, and 100%, maximum accumulation rates occurred at years 14, 18, and 19, respectively. Similarly, maximum accumulation rates declined as carbon legacies increased (Figure 4d).

#### Effects of landscape age-structure

**Figure 5:**
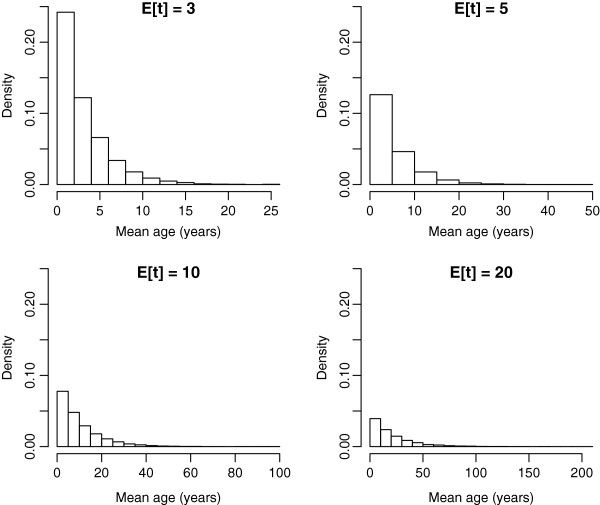
**Histograms of the hypothetical landscapes produced by randomly sampling exponential distributions with mean age *E*** **[*t*] = 3, 5, 10, and 20 years.**

Hypothetical landscapes produced by randomly sampling exponential probability distributions with different mean age, produced in all cases a large number of young landscape units (Figure 5). For all mean landscape ages, the amount of landscape units in early successional stages was always higher than the amount of units in late successional stages. Maximum age of landscape units increased with mean landscape age.

**Figure 6:**
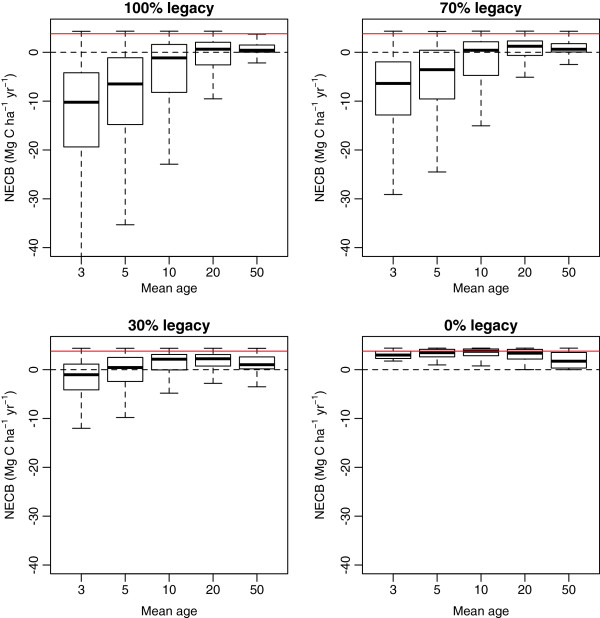
**Net ecosystem carbon balance (*NECB*) predicted under different assumptions of carbon legacies after land-use change and mean age of the landscape.** The horizontal red line in each panel represents the carbon accumulation rate used by [6] to predict carbon accumulation rates in tropical America.

In the presence of carbon legacies, carbon was predominantly released in landscapes with a higher proportion of young forests. Landscapes with mean ages below 10 years, can be important sources of carbon to the atmosphere depending on the degree of carbon legacies. However, the release of carbon generated by the legacies can be offset by carbon uptake in older landscape units if the amount of legacies is relatively small (< 30%) and the mean age of the landscape is relatively high (> 10 years old) (Figure 6).

Without the presence of carbon legacies, carbon accumulation rates are always positive in landscapes of any given age (Figure 6). On average, the maximum accumulation rates occur in landscapes with mean ages around 10 years. When the mean age of the landscape surpasses this maximum rates, the rates of carbon accumulation decline.

The rates of carbon accumulation used to calculated tropical forest regrowth in the Americas [6] were always above the rates we found with our empirical model for different levels of carbon legacies and mean landscape age; except, for the situation of no legacies and a mean landscape age of 10 years (Figure 6).

## Discussion

Although the data and the empirical model we present here were derived for one single location from the enormous area of tropical regrowing forests, the good agreement of our estimates with other sites suggests that general trends of total carbon accumulation may exists for regrowing tropical forests at larger scales. Aboveground biomass accumulation in global and tropical secondary forests appears to be highly predictable [7,26,27], so total carbon accumulation may also be possible to predict from large-scale environmental variables as more information becomes available.

Despite the limitation of our data for large-scale inferences, our empirical model can be used to explore possible effects of different assumptions in the calculation of carbon accumulation rates at the biome level. Three main assumptions are discussed below: 1) the use of tree aboveground biomass as a surrogate for total accumulation rates, 2) the explicit inclusion of carbon legacies, and 3) the landscape age structure of regrowing forests.

### Tree aboveground biomass as surrogate for total carbon accumulation

A considerable number of studies have calculated accumulation rates of aboveground biomass in regrowing tropical forests (e.g., [28-37]). Less common however, is the estimation of total carbon accumulation rates that also include belowground and soil pools (however see, [7,21,22]). In this study we report total carbon accumulation rates that can help to reduce uncertainties associated with the carbon balance of tropical regrowing forests [6].

Our results suggest that the contribution of tree aboveground biomass to total carbon accumulation is very important in the late stages of succession. In early stages however, the contribution of fine roots and total dead mass can be important (Figures 1 and 2). Because most of the carbon in aboveground tree biomass accumulates in the trunk, the contribution of aboveground biomass to total carbon accumulation is only significant until the trees reach a stage in which trunk biomass is considerably larger than the carbon accumulation in leaves and roots. In fact, fine root biomass does not increase over time in our study site, in agreement with previous studies which suggests that the amount of fine roots at the ecosystem level recovers quickly after disturbances [4,38].

Other components of aboveground biomass such as palms and herbaceous vegetation contribute a very small fraction compared to the contributions of tree aboveground biomass. Given the difficulties and costs associated with measuring these components in the field, it would be possible to exclude them from estimations of total carbon accumulation without incurring in important underestimation.

However, the exclusion of other ecosystem carbon pools such as coarse- and fine-root biomass as well as fine litter and coarse woody debris, can lead to important underestimation of total carbon accumulation. These pools can contribute between 50 and 5% of the total mass (*TM*) or between 20 and 1% of total carbon stocks (*TCS*), depending on succession age (Figure 2).

In particular, total dead mass can be an important component of the carbon flux in regrowing forests. Although dead material decomposes and emit carbon to the atmosphere, it also accumulates carbon over time (Figure 2), being an important component of total carbon accumulation in regrowing forests [13].

After total aboveground biomass, the second largest carbon pool in the secondary forests we studied was soil carbon. However, in the studied plots we were not able to observe carbon accumulation trends in the soil component. This suggests that, as a carbon pool, soils are an important component of these regrowing forests, but in terms of carbon accumulation rates the contribution of the soils is negligible.

Lack of carbon accumulation in bulk soils during secondary succession has been reported previously [39-41]. However, a significant number of studies show positive carbon accumulation rates in soils after conversion from pasture or grassland to forest [42,43]. Isotope analyses have revealed that soil carbon in secondary forests is the net result of inputs from the newly stablished forest minus losses of carbon from the previous land use [7,41]. The amount of inputs in the secondary forests of the study area are significantly lower than inputs in undisturbed forests, while the amount of outputs through soil respiration are relatively high. This situation creates a net carbon balance in the soil close to zero [44].

In other ecosystems, the transition from grassland and cropland to secondary forest may result in a gain of soil carbon of up to 50 Mg C ha^-1^[42]. Assuming this recovery occurs between the first 50 to 100 years of succession, the annual rates of carbon accumulation could be between 0.5 to 1.0 Mg C ha^-1^yr^-1^, and could be even larger than accumulation rates in *TBB* and *TDM*. However, rates of carbon accumulation in soil vary widely across the tropics and depend on multiple factors such as precipitation and soil texture and minerology [43].

In summary, accumulation rates in tree aboveground biomass can only give good approximations to total carbon accumulation rates in late stages of forest succession and where soil carbon does not recover to the levels of primary forests. In all other cases, carbon accumulation in tree aboveground biomass probably underestimates total carbon accumulation rates in considerable proportions.

### Carbon legacies

Although in the secondary forest landscape we studied there was no evidence of C legacies due to its long history of land use, we were interested in exploring the C consequences of such legacies in the hypothetical case they had been present. In addition, large-scale carbon budgets calculate the net C balance between deforestation and regrowth implicitly including these C legacies; however, it is difficult to assess the effects of different changes in land use on the temporal trend of C recovery without the explicit account of these legacies.

Carbon legacies can vary in importance depending on the type of disturbance or previous land use. Extreme weather events such as hurricanes [15] or storms [16] can cause significant tree mortality over large areas. After these events, a large percentage of carbon is transferred from live to dead pools, with 100% of the dead material remaining in situ. In these cases, the regrowing forests after disturbance will likely behave as carbon sources during the first years of forest recovery [45].

The type and amount of carbon legacies after the abandonment of agricultural or pasture lands is more complex. After harvesting, crop residues remain in situ acting as a carbon legacy. However, the amount of this carbon legacy varies substantially depending on the type of crop; cereals and sugar crops producing the highest amount of residue in comparison to legumes and oil crops [18].

In forests recovering after pastures, other biological legacies may have more important effects on carbon accumulation than just carbon legacies. Pastures can significantly delay the recovery process due to a number of biological factors such as the rapid growth of herbs and ferns [28]. In our study area, where pasture was the dominant previous land use, we did not observe significant lags in the recovery of carbon stocks. Similarly, other studies have not found differences in carbon accumulation rates after pasture compared to agriculture [27]. Carbon accumulation rates after pastures can be complex, resulting from the interaction of many factors such as species composition, seed source and dispersers, and degradation of soil properties [28].

Small and large-scale timber extraction can also have important impacts on the amount and type of carbon legacies. Forestry activities rarely extract 100% of the harvested trees, leaving in situ a considerable amount of slash that acts as carbon legacy. Keller et al. [17] report that conventional logging systems in the Amazon can increase the amount of coarse woody debris by 95% above the background levels in undisturbed forests. In addition to this increase, other slash and fine debris represent an important amount of legacy carbon that can significantly delay positive accumulation rates in regrowing vegetation.

### Landscape age structure

Our simulations showed that depending on succession age and amount of carbon legacies, the magnitude and direction of ecosystem carbon fluxes can differ significantly. Landscapes are usually mosaics of patches with different ages and land-use/disturbance histories [9]. Therefore, it is very important to study carbon sources and sinks accounting for this heterogeneity in landscape structure.

Previous studies have found that the age of secondary forest patches in the Amazon can be well represented by exponential density functions with mean age between 4 and 5 years [11]. Although age distributions can vary geographically across secondary tropical forests due to different economic, social and political factors, it is likely that mean ages are low as reported for the Brazilian Amazon. This relatively high proportion of forests in very early stages of succession has two important implications: 1) carbon legacies probably dominate the behavior of carbon fluxes in these early stages, 2) tree aboveground biomass does not contribute as much to total carbon accumulation as in later succession stages.

In the presence of carbon legacies, these relatively high proportion of forests in early successional stages suggests that important portions of anthropogenic landscapes in the Amazon may be actually acting as carbon sources rather than carbon sinks.

This relative small mean age of tropical forest landscapes and the results from our simulations also suggest that the contribution of tree aboveground biomass to total accumulation rates is not as high as it could be in other landscapes with higher mean ages. Therefore, the inclusion of belowground, dead, and soil carbon pools in calculations of carbon accumulation rates should give more priority to carbon accumulation in these other pools.

### Implications for large-scale carbon budgets

Tropical forest regrowth has been included implicitly in large scale estimations of land-use change [8], but there is renewed interested in separating it from deforestation emissions [6]. To reduce uncertainties and biases, and produce more accurate estimations, it is important to specifically account for carbon accumulation rates in belowground biomass, dead material, and soil carbon, in addition to aboveground biomass. This can potentially reduce underestimations of carbon accumulation between 20 and 50%. In addition, large scale budgets should also consider with detail the amount of carbon legacies after different land uses, and the age-structure of different landscapes.

Estimations of carbon emissions from tropical land-use change implicitly account for carbon legacies, forest age, and belowground carbon stocks (e.g., [46-50]). However, more detailed representations of these processes can help to understand key processes related to previous land use and carbon accumulation over time. For example, our simulations showed that carbon legacies control the period of time in which secondary forests act as carbon sources, the maximum accumulation rate that can be achieved during the entire successional process, and the time required to reach this maximum accumulation rate. Whether regrowing forests after different land uses with different carbon legacies may behave as carbon sources or sinks, is a question that cannot be answered with previous carbon accounting schemes. These type of questions are important for forest management and should help not only to produce estimates of the carbon consequences of land use, but also to devise possible management strategies to reduce carbon emissions.

The carbon accumulation rate of 3.8 Mg C ha^-1^yr^-1^ recently used to estimate tropical forest regrowth in the Americas [6], agrees well with our estimations of carbon accumulation in landscapes with mean age of ∼10 years and without the influence of carbon legacies (Figure 6). Whether these assumptions hold for all regrowing tropical forests in the Americas is uncertain, therefore new research efforts to quantify secondary forest age-structure would help to produce more accurate carbon budgets.

## Conclusions

A chronosequence of total carbon accumulation in a secondary forest landscape where pasture was the dominant previous land use, showed that carbon accumulates over time following a sigmoidal curve, reaching the maximum carbon levels between 80 and 100 years. Soil carbon stocks did not recover to levels of the undisturbed forests however, suggesting that previous land use severely modified the capacity to recover after anthropogenic disturbance.

A statistical fit to the data showed that this carbon accumulation is highly predictable, with maximum accumulation rates at around 9 years after the start of the successional process. The contribution of different pools varied over time, with aboveground biomass representing a small proportion in early stages but being the dominant carbon pool in late stages of forest succession.

The empirical model derived from the observations in this andean tropical forest landscape, performed remarkably well predicting carbon accumulation in other chronosequences in the Amazon basin and Costa Rica. This suggests that the process of total carbon accumulation is highly predictable and better models can be developed in the future as more data becomes available to predict carbon accumulation at the continental scale. In addition, this model can be used to explore different assumptions in calculating carbon accumulation rates at larger scales.

Simulation results using our empirical model allowed us to reach three important conclusions: 1) tree aboveground biomass is only a good surrogate for total carbon accumulation in late stages of forest succession. In the Brazilian Amazon, secondary forest landscapes have a mean age between 4 and 5 years [11] and young landscapes may also occur frequently in the tropics, therefore estimates based only on aboveground biomass probably underestimate total carbon accumulation. 2) Carbon legacies from previous land use can have important effects on the magnitude and direction of carbon fluxes in secondary forests. In the presence of legacies, secondary forests can act as carbon sources to the atmosphere, can decrease the maximum accumulation rate, and delay the time at which this maximum rate is reached. 3) Mean landscape age determines the magnitude and the direction of carbon fluxes. Without the presence of legacies, landscape age determines the strength of the carbon sink. In the presence of legacies, landscape age determines whether a tropical forest landscape is acting as a carbon source or a sink.

## Methods

### Data collection and study site

In this study, we used data collected in secondary and primary forests of the Porce region of Colombia (6°45’ 37” N, 75°06’ 28” W). Mean annual temperature and precipitation in this region are reported as 22.7°C and 2078 mm, respectively. The main soil orders are Entisols and Inceptisols, with an average bulk density of 1.3 Mg m^-3^.

Secondary forests in the region were established in the late 1980s and 1990s after the design and construction of a series of hydroelectric projects that required the abandonment of pasture land to create a buffer zone surrounding the dams. Original deforestation of the area dates back to the mid 20th century when the majority of the area was converted to cattle pastures and a minor proportion to agriculture [51].

Previously, we had reported for this area total carbon stocks for primary and secondary forests [52], rates of carbon uptake and release in primary forests [53], rates of above- and below-ground carbon accumulation in secondary forests [51], soil carbon balance [44], and changes in forest structure and composition along the successional sequence [54]. Here, we compile these previous results, add new data, and present estimates of total carbon accumulation rates across all carbon pools in the secondary forests.

Between 1999 and 2001 we established 110 permanent plots in primary and secondary forests where we measured all trees and palms with diameter at breast height *D *> 1 cm. Between 2005 and 2006, 33 additional plots were established in secondary forests [51]. Local aboveground biomass equations were developed for trees in the two forest types as well as allometric equations for palms and coarse roots [52]. We also harvested total above and belowground biomass in recently (3-5 years) abandoned pastures.

Soil carbon was measured in all plots at two depths 0-15 and 15-30 cm by taking samples for bulk density and composite samples at each plot for percent carbon content determination [52]. Fine root biomass (< 0*.*5 cm in diameter) was sampled at the same depths in all plots extracting 3 soil cores (8 cm in diameter x 15 cm long) per plot.

For a subset of 33 plots in the secondary forests, we estimated the age since land abandonment using a combination of techniques: 1) interviews with local inhabitants, 2) tree-ring analysis in species with known annual rings, 3) radiocarbon dating of trees with average diameter, and 4) land-cover sequences from aerial photography and satellite images. With these ages we were able to ensemble a set of plots that form a chronosequence that spans 36 years, from recently abandoned pastures to well developed successional forests. Additional details about the calculation of plot age in this chronosequence are presented in [51].

For each plot we determined total aboveground biomass (*TAGB*) as the sum of aboveground tree biomass, aboveground palm biomass, and herbaceous vegetation (*D *< 1 cm). Total belowground biomass (*TBB*) was calculated as the sum of fine and coarse roots for each plot. Total dead mass (*TDM*) as the sum of fine litter and coarse woody debris. Soil organic carbon (*SOC*) as the sum of the estimates at 0-15 and 15-30 cm depth. Total carbon stocks (*TCS*) are then the sum of all these components for each plot as

(2) $$\text{TCS}=p_{c}\left(\text{TAGB},+,\text{TBB},+,\text{TDM}\right)+\text{SOC},$$

where *p*_*c *_is the proportion of carbon in dry organic matter (DW). In our case, we used *p*_*c *_= 0*.*45, estimated from measurements of different pools at the site [52].

Ecosystem carbon fluxes were calculated as the annual difference in *TCS*, which corresponds to the concept of Net Ecosystem Carbon Balance [55]

(3) $$\text{NECB}=\frac{\Delta \text{TCS}}{\Delta t}.$$

### Model fits

We used common empirical models [19,51,56] for predicting biomass and carbon accumulation in all pools along the successional sequence. Relationships between the dependent variable *Y * and age *t* were fit using the von Bertalanffy growth model [57]

(4) $$Y=Y_{\text{max}}{\left(1,-,\beta_{0},\exp,\left(-,\beta_{1},\;,t\right)\right)}^{\beta_{2}},$$

and the logistic equation

(5) $$Y=\frac{Y_{\text{max}}}{\left(1,+,\beta_{1},\exp,\left(\beta_{2},\;,t\right)\right)},$$

where *β*_0_, *β*_1_, and *β*_2_ are empirical coefficients, and *Y*_*max *_the maximum average value of the dependent variable.

The values of *Y*_*max*_ for the three models were set as constants in fitting the regression models, using the average values for these pools measured in the primary forests of the region: *TAGB*_*max *_= 111*.*51, *TBB*_*max *_= 37*.*665, and *TDM*_*max *_= 6*.*615 Mg C ha^-1^[52].

### Simulations

We explored the effects of carbon legacies and landscape age-structure using simulations of carbon accumulation from the fitted models. Before running the simulations, we evaluated the performance of the model using an independent dataset of total carbon accumulation for an Amazon tropical forest [20,21], and a lowland tropical forest from Costa Rica [22].

We tested the effects of different levels of carbon legacies on TCS and NECB by performing simulations in which different amounts of C were present at the initiation of forest succession and decomposed over time. In particular, we tested the effects of leaving in situ 100, 70, 30 and 0% of the total mass (*TM *=* TAGB* + *TBB* + *TDM*) present under primary forest using the equation

(6) $$\begin{array}{ll} \;\text{TCS}(t)= & \text{TM}(t=0)\;e^{\left(-,k,\;,t\right)}+\text{TAGB}\left(t\right)+\text{TBB}\left(t\right)\\ & +\text{TDM}(t)+\text{SOC}(t). \end{array}$$

Decomposition of the legacy C (*TM*(*t *= 0)) was represented with a simple exponential model using the median decomposition rate *k* of 155 logs measured in Central Amazonia [24]. We used here the complete data set from [24], which is publicly available from the Oak Ridge National Laboratory Distributed Active Archive Center (ORNL DAAC) [25].

The effects of landscape age-structure on carbon fluxes were tested by producing hypothetical landscape configurations following an exponential probability distribution [11]

(7) $$p\left(t\right)=\lambda e^{-\lambda t}$$

where *t* is the age of a landscape unit and its probability is given by *p*(*t*) with an expected age given by *E*[*t*] = 1/*λ*.

We produced hypothetical landscapes by sampling sets of 1000 random numbers from an exponential distribution with mean ages *E*[*t*] = 3, 5, 10, 20, and 50 years. To each set of 1000 landscape units, we calculated the values of NECB predicted by the empirical model under different assumptions of carbon legacies. To assess the uncertainty introduced by the value of the decomposition rate, we applied different values of *k* sampled randomly from the set of 155 logs using a Monte Carlo procedure.

The code and data to reproduce all results from this analysis are provided in the supplementary material for verification and reuse (see Additional files 1, 2, and 3).

## Competing interests

The authors declare that they have no competing interests.

## Authors’ contributions

CAS conceived study, analyzed data, and wrote the paper. JIdV conceived the study and performed research, HIR performed research. All authors read and approved the final manuscript.

## Supplementary Material

Additional file 1 **PorcedB2012.csv.** Biomass (Mg DW ha^-1^yr^-1^) and soil carbon stocks (Mg C ha^-1^yr^-1^) of 33 permanent plots with their corresponding age. AGB: tree aboveground biomass (*D *> 1 cm), Palm: aboveground biomass of palms, HV: biomass of herbaceous and non-woody vegetation (*D *< 1 cm), CR: coarse root biomass (> 5 mm diameter), FR: fine root biomass (< 5 mm diameter), FL: fine litter, CWD: coarse woody debris, SC15: soil organic carbon to 15 cm depth, SC30: soil organic carbon to 30 cm depth. Click here for file

Additional file 2 **TCA_Porce.R.** R code to reproduce all graphics and simulations in the manuscript. Click here for file

Additional file 3 **Chambers_k.csv.** Database on coarse wood decomposition rates obtained from [25]. This is a short version of the dataset, included here only for the purpose to run the code provided as Additional file 2. Click here for file

## References

[B1] DeFries R Rudel T Uriarte M Hansen M Deforestation driven by urban population growth and agricultural trade in the twenty-first century Nat Geoscience 2010 3 3 178 181

[B2] Geist H Lambin E Proximate causes and underlying driving forces of tropical deforestation BioScience 2002 52 2 143 150

[B3] Morton D Defries R Randerson J Giglio L Schroeder W Van Der Werf G Agricultural intensification increases deforestation fire activity in Amazonia Glob Change Biol 2008 14 10 2262 2275

[B4] Brown S Lugo AE Tropical secondary forests J Trop Ecol 1990 6 1 1 32

[B5] Guariguata MR Ostertag R Neotropical secondary forest succession: changes in structural and functional characteristics Forest Ecol Manage 2001 148 1-3 185 206 10.1016/S0378-1127(00)00535-1

[B6] Pan Y Birdsey R Fang J Houghton R Kauppi P Kurz W Phillips O Shvidenko A Lewis S Canadell J A large and persistent carbon sink in the world’s forests Science 2011 333 6045 988 993 21764754 10.1126/science.1201609

[B7] Silver WL Ostertag R Lugo AE The potential for carbon sequestration through reforestation of abandoned tropical agricultural and pasture lands Restoration Ecol 2000 8 4 394 407 10.1046/j.1526-100x.2000.80054.x

[B8] Ramankutty N Gibbs HK Achard F Defries R Foley JA Houghton RA Challenges to estimating carbon emissions from tropical deforestation Global Change Biol 2007 13 1 51 66 10.1111/j.1365-2486.2006.01272.x

[B9] Bormann F Likens G Pattern and Process in a Forested Ecosystem: Disturbance, Development, and the Steady State Based on the Hubbard Brook Ecosystem Study 1994 New York: Springer

[B10] Neeff T de Alencastro Graca PM Dutra LV da Costa Freitas C Carbon budget estimation in Central Amazonia: Successional forest modeling from remote sensing data Remote Sensing Environ 2005 94 4 508 522 10.1016/j.rse.2004.12.002

[B11] Neeff T Lucas RM dos Santos JR Brondizio ES Freitas CC Area and age of secondary forests in Brazilian Amazonia 1978–2002: An empirical estimate Ecosystems 2006 9 4 609 623 10.1007/s10021-006-0001-9

[B12] Franklin JF Spies TA Pelt RV Carey AB Thornburgh DA Berg DR Lindenmayer DB Harmon ME Keeton WS Shaw DC Bible K Chen J Disturbances and structural development of natural forest ecosystems with silvicultural implications, using douglas-fir forests as an example Forest Ecol Manage 2002 155 1-3 399 423 10.1016/S0378-1127(01)00575-8

[B13] Harmon M Carbon sequestration in forests: addressing the scale question J Forestry 2001 99 4 24 29

[B14] Chambers JQ Fisher JI Zeng H Chapman EL Baker DB Hurtt GC Hurricane Katrina’s carbon footprint on U.S. Gulf Coast forests Science 2007 318 5853 1107 10.1126/science.1148913 18006740

[B15] Lugo AE Effects and outcomes of caribbean hurricanes in a climate change scenario Sci Total Environ 2000 262 3 243 251 10.1016/S0048-9697(00)00526-X 11087030

[B16] Negrón-Juárez R Chambers J Guimaraes G Zeng H Raupp C Marra D Ribeiro G Saatchi S Nelson B Higuchi N Widespread Amazon forest tree mortality from a single cross-basin squall line event Geophysical Res Lett 2010 37 16 L16,701

[B17] Keller M Palace M Asner G Pereira Jr R Silva J Coarse woody debris in undisturbed and logged forests in the eastern Brazilian Amazon Glob Change Biol 2004 10 5 784 795

[B18] Lal R World crop residues production and implications of its use as a biofuel Environ Int 2005 31 4 575 584 15788197 10.1016/j.envint.2004.09.005

[B19] Janisch J Harmon M Successional changes in live and dead wood carbon stores: implications for net ecosystem productivity Tree Physiol 2002 22 2-3 77 89 11830405 10.1093/treephys/22.2-3.77

[B20] Saldarriaga JG Recuperación de la selva de “Tierra Firme” en el alto río Negro Amazonia colombiana-venezolana Tropenbos-Colombia, Bogotá 1994

[B21] Saldarriaga JG West DC Tharp ML Uhl C Long-term chronosequence of forest succession in the upper rio negro of colombia and venezuela J Ecol 1988 76 4 938 958

[B22] Fonseca W a Rey Benayas JM Alice FE Carbon accumulation in the biomass and soil of different aged secondary forests in the humid tropics of Costa Rica Forest Ecol Manage 2011 262 8 1400 1408 10.1016/j.foreco.2011.06.036

[B23] Law B Turner D Campbell J Sun O Van Tuyl S Ritts W Cohen W Disturbance and climate effects on carbon stocks and fluxes across Western Oregon USA Glob Change Biol 2004 10 9 1429 1444

[B24] Chambers JQ Higuchi N Schimel JP Ferreira LV Melack JM Decomposition and carbon cycling of dead trees in tropical forests of the central Amazon Oecologia 2000 122 3 380 388 10.1007/s004420050044 28308289

[B25] Chambers JQ Schimel JP Nobre AD Higuchi N Ferreira LV Melack JM Trumbore SE LBA-ECO CD-08 Coarse wood litter respiration and decomposition, Manaus, Brazil Dataset 2009 10.3334/ORNLDAAC/911

[B26] Johnson CM Zarin DJ Johnson AH Post-disturbance aboveground biomass accumulation in global secondary forests Ecology 2000 81 5 95 1401

[B27] Zarin DJ Ducey MJ Tucker JM Salas WA Potential biomass accumulation in Amazonian regrowth forests Ecosystems 2001 4 7 658 668 10.1007/s10021-001-0035-y

[B28] Aide T Zimmerman J Herrera L Rosario M Serrano M Forest recovery in abandoned tropical pastures in Puerto Rico Forest Ecol Manage 1995 77 1-3 77 86 10.1016/0378-11279503576-V

[B29] Fehse J Hofstede R Aguirre N Paladines C Kooijman A Sevink J High altitude tropical secondary forests: a competitive carbon sink? Forest Ecol Manage 2002 163 1-3 9 25

[B30] Feldpausch T Rondon M Fernandes E Riha S Wandelli E Carbon and nutrient accumulation in secondary forests regenerating on pastures in central amazonia Ecol Appl 2004 14 sp4 164 176

[B31] Hughes R Kauffman J Jaramillo V Biomass, carbon, and nutrient dynamics of secondary forests in a humid tropical region of Mexico Ecology 1999 80 6 1892 1907

[B32] Hughes RF Kauffman JB Cummings DL Dynamics of aboveground and soil carbon and nitrogen stocks and cycling of available nitrogen along a land-use gradient in Rondônia, Brazil Ecosystems 2002 5 3 244 259 10.1007/s10021-001-0069-1

[B33] Silver WL Kueppers LM Lugo AE Ostertag R Matzek V Carbon sequestration and plant community dynamics following reforestation of tropical pasture Ecol Appl 2004 14 4 1115 1127

[B34] Steininger MK Net carbon fluxes from forest clearance and regrowth in the Amazon Ecol Appl 2004 14 4 S313 S322

[B35] Toky OP Ramakrishnan PS Secondary succession following slash and burn agriculture in North-Eastern India: I. biomass, litterfall and productivity J Ecol 71 3 735 745

[B36] Uhl C Jordan CF Succession and nutrient dynamics following forest cutting and burning in Amazonia Ecology 1984 65 5 1476 1490

[B37] Uhl C Buschbacher R Serrao EAS Abandoned pastures in Eastern Amazonia. I. Patterns of plant succession J Ecol 1988 76 3 663 681

[B38] Berish CW Ewel JJ Root development in simple and complex tropical successional ecosystems Plant Soil 1988 106 1 73 84 10.1007/BF02371197

[B39] Cleveland CC Townsend AR Schmidt SK Constance BC Soil microbial dynamics and biogeochemistry in tropical forests and pastures, southwestern Costa Rica Ecol Appl 2003 13 2 314 326

[B40] Marín-Spiotta E Sharma S Carbon storage in successional and plantation forest soils: a tropical analysis Glob Ecol Biogeography 2012 10.1111/j.1466-8238.2012.00788.x

[B41] Marin-Spiotta E Silver W Swanston C Ostertag R Soil organic matter dynamics during 80 years of reforestation of tropical pastures Glob Change Biol 2009 15 6 1584 1597

[B42] Don A Schumacher J Freibauer A Impact of tropical land-use change on soil organic carbon stocks–a meta-analysis Glob Change Biol 2011 17 4 1658 1670

[B43] Powers JS Corre MD Twine TE Veldkamp E Geographic bias of field observations of soil carbon stocks with tropical land-use changes precludes spatial extrapolation Proc Natl Acad Sci 2011 10.1073/pnas.1016774108 PMC3076837 21444813

[B44] Moreno FH Oberbauer SF Bravo F, Jandl R, LeMay V, Gadow K Dynamics of soil carbon in primary and secondary tropical forests in Colombia Managing Forest Ecosystems: The Challenge of Climate Change, Managing Forest Ecosystems, vol 17 2008 Netherlands: Springer 283 296 10.1007/978-1-4020-8343-3-16

[B45] Fisher J Hurtt G Thomas R Chambers J Clustered disturbances lead to bias in large-scale estimates based on forest sample plots Ecol Lett 11 6 554 563 18373680 10.1111/j.1461-0248.2008.01169.x

[B46] Achard F Eva H Mayaux P Stibig H Belward A Improved estimates of net carbon emissions from land cover change in the tropics for the 1990s Global Biogeochemical Cycles 2004 18 2 1 11 10.1029/2003GB002142

[B47] DeFries R Houghton R Hansen M Field C Skole D Townshend J Carbon emissions from tropical deforestation and regrowth based on satellite observations for the 1980s and 1990s Proc Natl Acad Sci 2002 99 22 14,256 10.1073/pnas.182560099 PMC137871 12384569

[B48] Hirsch A Little W Houghton R Scott N White J The net carbon flux due to deforestation and forest re-growth in the Brazilian Amazon: analysis using a process-based model Global Change Biol 2004 10 5 908 924

[B49] Houghton R Revised estimates of the annual net flux of carbon to the atmosphere from changes in land use and land management 1850–2000 Tellus B 2003 55 2 378 390

[B50] Houghton R Skole D Nobre C Hackler J Lawrence K Chomentowski W Annual uxes of carbon from deforestation and regrowth in the Brazilian Amazon Nature 2000 403 301 304 10659847 10.1038/35002062

[B51] del Valle J Restrepo H Londoño M Recuperación de la biomasa mediante la sucesión secundaria, cordillera central de los Andes, Colombia Revista de Biología Tropical 59 3 1337 1358 22017137

[B52] Sierra CA del Valle JI Orrego SA Moreno FH Harmon ME Zapata M Colorado GJ a A Herrera M Lara W Restrepo DE Berrouet LM Loaiza LM Benjumea JF Total carbon stocks in a tropical forest landscape of the Porce region, Colombia Forest Ecol Manage 2007 2-3 299 309 10.1016/j.foreco.2007.03.026

[B53] Sierra CA Harmon ME Moreno FH Orrego SA del Valle JI Spatial and temporal variability of net ecosystem production in a tropical forest: testing the hypothesis of a significant carbon sink Glob Change Biol 2007 13 4 838 853 10.1111/j.1365-2486.2007.01336.x

[B54] Yepes A del Valle J Jaramillo S Orrego S Recuperación estructural en bosques sucesionales andinos de Porce (Antioquia, Colombia) Revista de Biologí a Tropical 2010 58 1 427 445 20411733

[B55] Chapin FS Woodwell GM Randerson JT Rastetter EB Lovett GM Baldocchi DD Clark DA Harmon ME Schimel DS Valentini R Reconciling carbon-cycle concepts, terminology, and methods Ecosystems 2006 9 7 1041 1050 10.1007/s10021-005-0105-7

[B56] McMahon S Parker G Miller D Evidence for a recent increase in forest growth Proc Natl Acad Sci 2010 107 8 3611 3615 20133710 10.1073/pnas.0912376107 PMC2840472

[B57] von Bertalanffy L Quantitative laws in metabolism and growth Q Rev Biol 1957 32 3 217 231 13485376 10.1086/401873

